# Design of the smart home system based on the optimal routing algorithm and ZigBee network

**DOI:** 10.1371/journal.pone.0188026

**Published:** 2017-11-13

**Authors:** Dengying Jiang, Ling Yu, Fei Wang, Xiaoxia Xie, Yongsheng Yu

**Affiliations:** 1 School of Science, Wuhan University of Technology, Wuhan, Hubei, China; 2 Physical Science and Technology Department, Central China Normal University, Wuhan, Hubei, China; 3 Department of Mathematics & Statistics, Idaho State University, Pocatello, Idaho, United States of America; 4 State Key Laboratory of Silicate Materials for Architectures, Wuhan University of Technology, Wuhan, Hubei, China; Chongqing University, CHINA

## Abstract

To improve the traditional smart home system, its electric wiring, networking technology, information transmission and facility control are studied. In this paper, we study the electric wiring, networking technology, information transmission and facility control to improve the traditional smart home system. First, ZigBee is used to replace the traditional electric wiring. Second, a network is built to connect lots of wireless sensors and facilities, thanks to the capability of ZigBee self-organized network and Genetic Algorithm-Particle Swarm Optimization Algorithm (GA-PSOA) to search for the optimal route. Finally, when the smart home system is connected to the internet based on the remote server technology, home environment and facilities could be remote real-time controlled. The experiments show that the GA-PSOA reduce the system delay and decrease the energy consumption of the wireless system.

## 1. Introduction

The smart home has gained widespread attentions due to its flexible integration into everyday life. When the home environment and facilities are connected to a remote control device by the wireless sensor network, the smart home can monitor the home environment conveniently and remote control the equipment, such as the home appliance socket, lighting, temperature, humidity, smoke, infrared, magnetic door, etc. [1–2]

Nowadays, the smart home aroused general research interest. Some studies focus on the energy consumption of the entire home system and commit to reducing energy consumption with ensuring that the home environment is comfortable [3, 4]. A multi-objective mixed integer nonlinear programming model is used for optimal energy expand in a smart home, and the balance between energy saving and a comfortable lifestyle is considered in [5]. A mixed-integer nonlinear programming model is used to minimize the cost by shifting different types of appliances to optimal periods during the day, while the grid’s technical constrains and the customers’ preferences are satisfied. The reducing of greenhouse gas emission is also considered in [6]. [7] studies the energy management strategies and the economy of smart homes when renewable energy and energy storage are integrated, and use a stochastic energy management with plugged-in electric vehicle energy storage and photovoltaic array, where the electric cost is far less than the non-optimal control case. [8] designs a nonlinear predictive energy management strategy for the identical building with a rooftop photovoltaic system and second-life lithium-ion battery energy storage, and introduces an ANN to forecasting the load for the system.

While some other researches focus on how to generate a wireless network and how to select routing. To construct such a wireless network, the wireless modules will be previously embedded in the related devices. Here we pay attention to the energy consumption of the wireless nodes and the network’s delay. For this purpose, the use of a low-cost network and an optimal routing approach will benefit.

The traditional smart home system usually uses Wi-Fi or Bluetooth to generate a wireless network. Comparing to them, a ZigBee network is more convenient and efficient. ZigBee is based on the IEEE 802.15.4 standard. As shown in Table 1, it has many advantages, such as ad-hoc network, low cost, low power, low end-to-end delay etc. [9]

**Table 1:** Wireless network comparison [10].

Characteristic	Bluetooth	Wi-Fi	ZigBee
Distance	10m	50m	50–1600m
Max number of cell	8	2007	>65000
Power supply	Days	Hours	Years
Linking time	Up to 10s	Up to 3s	30ms
Cost of terminal unit	Low	High	Low
Ease of use	Normal	Hard	Low

ZigBee network can service far more nodes than Wi-Fi and Bluetooth. However, if lots of nodes join the network, it will be more complex and even lead to data loss. The optimal routing algorithm will overcome these defects and decrease the energy consumption, therefore prolong the service life of the nodes [11–12].

As for the routing algorithm, the Genetic Algorithm (GA) can converge to the globally better individuals though it is not very fast, while the Particle Swarm Optimization Algorithm (PSOA) has a fast convergence rate but sometimes it converges to a local optimal point. We introduce a GA-PSOA method to improve the energy consuming of network nodes and the response rate of the smart system [13–14].

Our main contribution is to design a GA-PSOA algorithm to search for the globe optimal routing with a fast speed, which improves the decreasing of energy expand and reduces the system delay.

The rest of the paper is organized as: the section 2 shows the materials and methods used in the system framework, and explains how to design the ZigBee nodes. The section 3 introduces the routing algorithm and it is verified by simulation, the section 4 puts up with the design of remote control. Conclusions are provided in the last section.

## 2. Framework and ZigBee node

In a smart home system, wireless modules will be added to each household devices and sensors, which are responsible for controlling the devices or collecting the household environment data. In this part, we use special hardware to generate a framework of smart system and show how to set a ZigBee node.

### 2.1 The framework of system

At first the wireless module CC2530 is installed in each item, which will be taken as a node in the wireless network. Then a ZigBee node will be selected as the coordinator, and a network will be built, to which each wireless module automatically joins by Z-Stack protocol. The coordinator connects Cortex-A8 through a serial port, and Cortex-A8 will achieve ZigBee protocol and TCP/IP protocol conversion. Finally, family router links the Cortex-A8 and the remote server, and then the remote control devices can access to the household equipment.

Moreover, ZigBee uses an ad-hoc network and the gateway can automatically restore the connection with the remote server in case of power off or network being disconnected, which will keep a persistent connection in the network to ensure the communications with the socket long connection mode.

**Fig 1:**
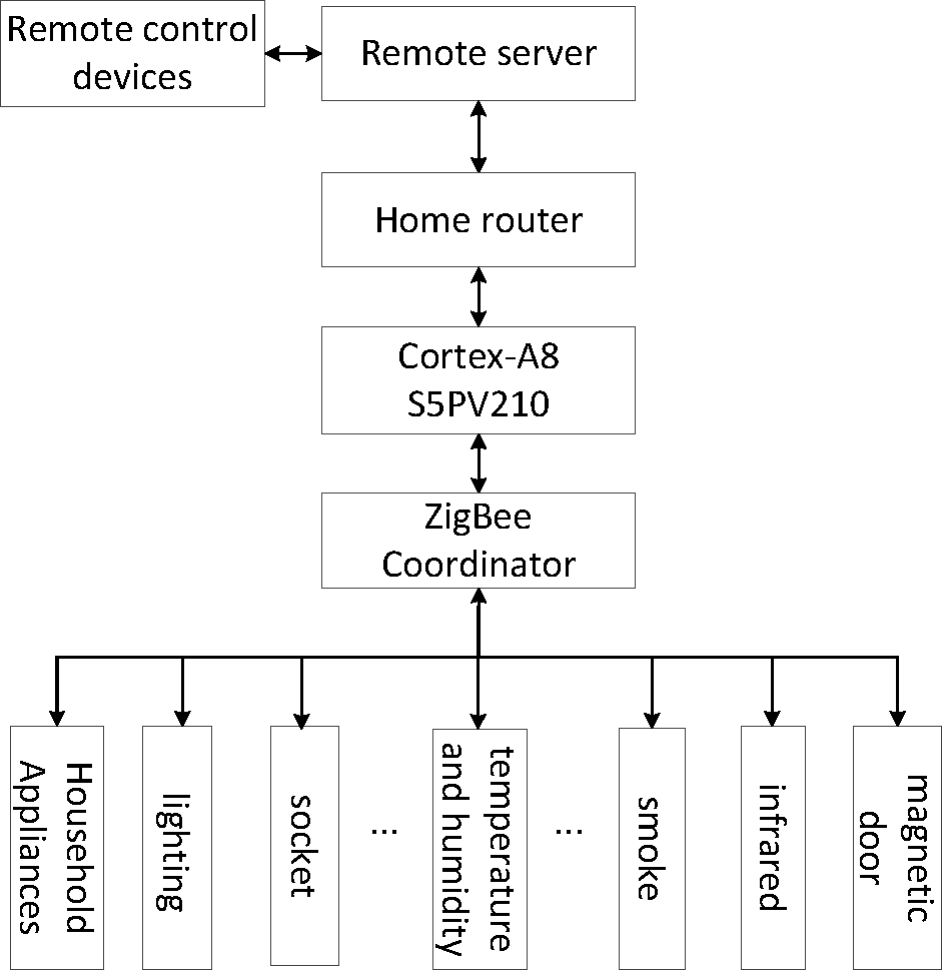
The framework of the system.

The basic framework of the system is shown in Fig 1.

There are three basic functions in the smart home system, remote control, query and alarm. The remote control client uses computers or mobile devices to send the corresponding control commands. When the ZigBee coordinator receives the instructions transferred from the remote server, the home router and the Cortex-A8 will judge the commands and forward them to the corresponding terminal devices. Then terminal devices call a function to parse instructions and perform the control commands. If it is a queried command, the sensor will collect the information and return the data to the ZigBee coordinator, which will send the data back to the remote control equipment. If the household sensor detects the abnormal situation, it will trigger the alarm system and send messages to the coordinator, as a result, the remote control equipment will receive the alarm message.

### 2.2 The ZigBee nodes

**Fig 2:**
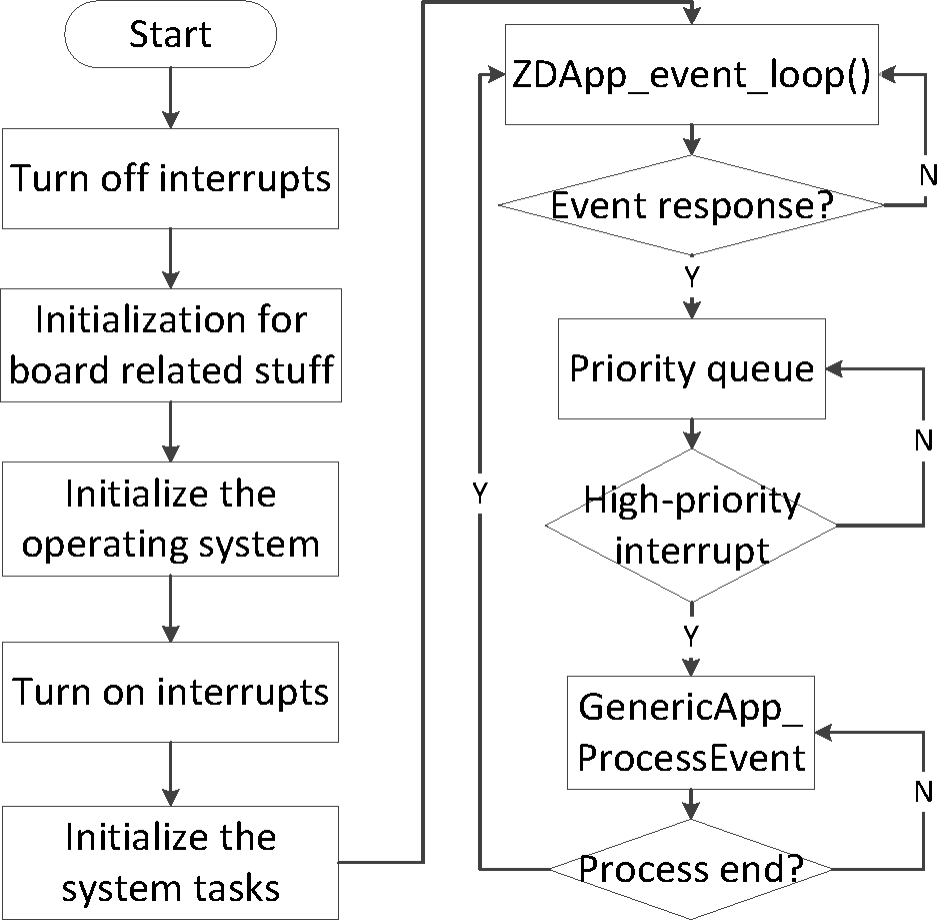
The ZigBee event handling process.

ZigBee nodes use Z-Stack protocols to form an ad-hoc network, which determines the occurrence of event by polling mechanism, and handles multiple events based on their priority. Furthermore, when the event processing completed, the system goes into sleep mode. Moreover, if any event occurs, the system will be waken up and run into the interrupt service routine to handle the event. The process of events handling is shown in Fig 2.

**Fig 3:**
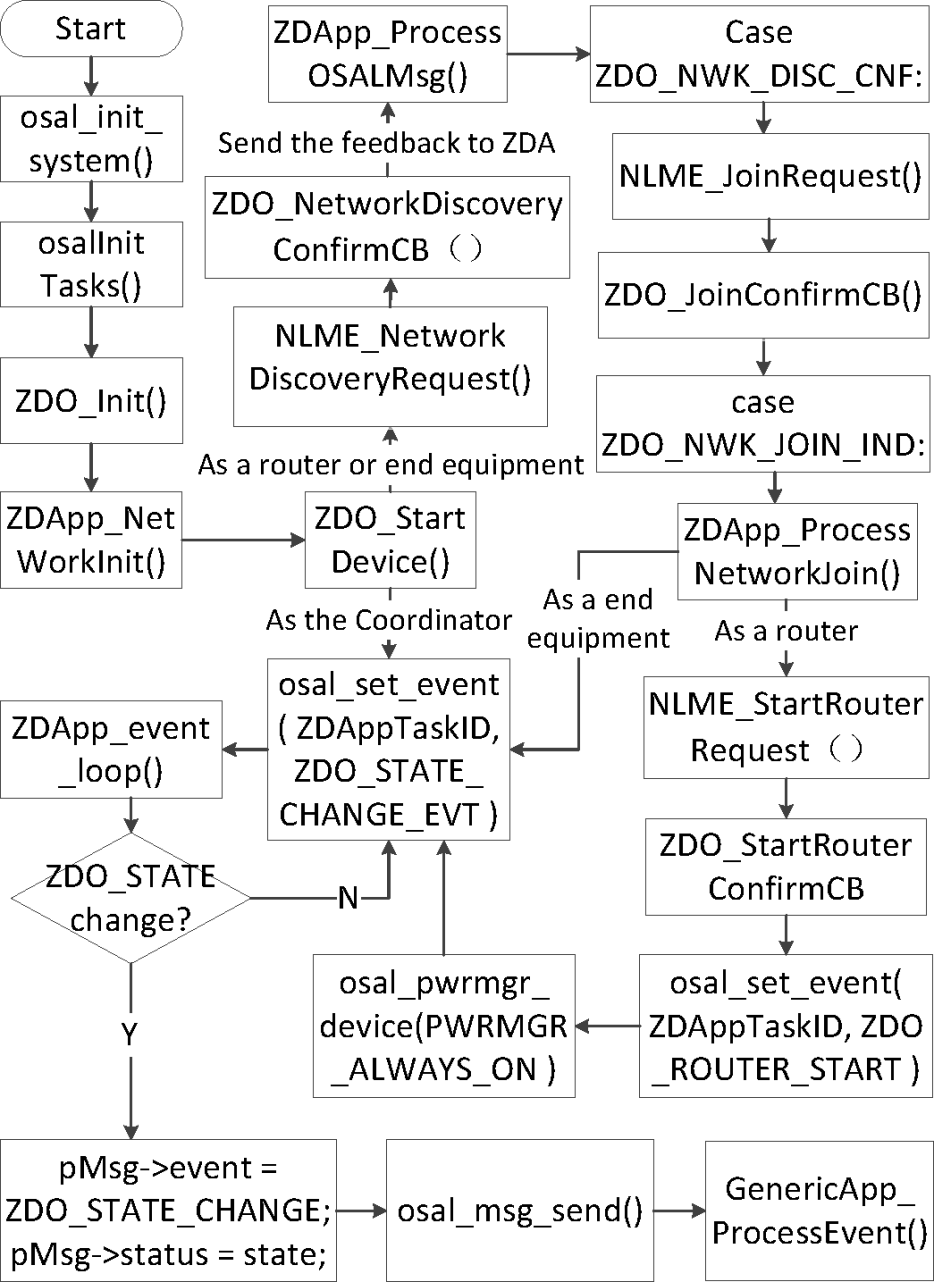
The process of ZigBee Ad-hoc network.

A ZigBee wireless network contains three different types of nodes, the coordinator, router and end equipment. In the Z-Stack protocol [15], APL layer, ZDO layer, APS layer and network layer are operated to initialize the ZigBee network, format the coordinator, manage the end devices, and join the routers into the network. The process of the ZigBee network’s formation and management is shown in Fig 3. At first, the system will initial the nodes, then identify all the nodes, if a node is coordinator, it will be initialized into the event set, if it is a router or an end equipment, it will be added into the NLME (Network layer management entity) system, which includes adding and leaving, addressing, receiving control, etc. Then if a node is a router, it will be added into the routing subsystem.

As for the program with ZigBee nodes, IAR Embedded Workbench IDE is usually used to design the programs of the coordinator, routers and end devices. When the node is identified as coordinator powered, the coordinator will automatically set up a wireless network. The routers will play a key role in routing and remote accessing, and devices will provide many functions, as they are equipped with all kinds of sensors. When routers and devices join the wireless sensor networks, the coordinator is responsible for managing those nodes, which to handle the incidents and remote requests.

## 3. The routing algorithm of ZigBee ad-hoc network

The quality of routing affects the system delay and energy consuming. Comparing to the general approach, the GA can achieve a global optimal goal, but it is not very fast, while the particle swarm optimization algorithm (PSOA) converge quickly, so we design a GA-PSOA method to utilize both sides’ advantage and then we will verify it through simulation.

### 3.1 Route analysis

With the development of smart home, the coverage area of ZigBee ad-hoc network gradually increase, and there will be more end devices joining the network. However, only one coordinator can exist in a ZigBee network. To ensure the remote real-time control, the number of routing node will increase, and then the routing path will become more complex. Therefore, adopting the appropriate routing algorithm becomes particularly important. Good routing will improve the performance of the system such as ensuring the communication quality, shortening the time delay, reducing the energy consumption of the nodes, etc.

In ZigBee networks, the traditional AODVjr routing algorithm creates routing on-demand, which provides a flexible lookup feature and improves the searching efficiency of the protocol, but it could cause flooding effect, which will lead to the increase of network energy consumption. The improved genetic algorithm has the ability of global searching, which can overcome the shortage of AODVjr algorithm and search for the optimal route [16]. However, the convergence speed of the genetic algorithm is usually slow, which increases the time delay of information transmission. As a result, if the genetic algorithm is combined with the PSOA which has the ability of fast convergence [17], it will further improve the searching ability of the network. We uses hybrid Genetic Algorithm-Particle Swarm Optimization Algorithm (GA-PSOA) to search for the optimization route [18,19], which can improve the performance of routing path selected. During the communications, if the best route exists in the current routing table, it will be selected, otherwise the system will search for the globally better routes based on the genetic algorithm, and then search the global best route through PSOA.

### 3.2 The design of routing algorithm

For a complex smart home system, numbers of ZigBee end equipment and sensors are randomly distributed in the family generally. Z-Stack protocol is used to form a wireless sensor network, and all the nodes will join the network, then we can use GA-PSOA to search for the global optimization route. The process is represented as follows.

Step 1: If a session start in a ZigBee network, and there is no best route in the routing table, the appropriate parameters of GA need to be set, where *m* routes from the source node to the destination node are recorded for *m* individuals in the population of the genetic algorithm.
Step 2: The adaptive value *f* is calculated by the fitness function in the population. Then the excellent individuals with the biggish fitness value will be selected for genetic operation, such as crossover and mutation, where the crossover probability and the mutation one can be controlled by *P*_*c*_ and *P*_*v*_ as Eq (2) and Eq (3).
Step 3: When the iteration of GA meets the criteria, excellent individuals will be selected for the step 4. Otherwise, continue to the step 2.
Step 4: To initialize parameters of PSOA, the excellent individuals will be optimized by PSOA. In every iteration, each particle records its optimal solution *P*_*b*_ and *P*_*g*_ searched by particle swarm. Particles will update the best path through the study of their history, and the information shared in the population.
Step 5: The step 4 will continue repeating until the result meets the convergence conditions, then the globally optimal solution will be the output.

**Fig 4:**
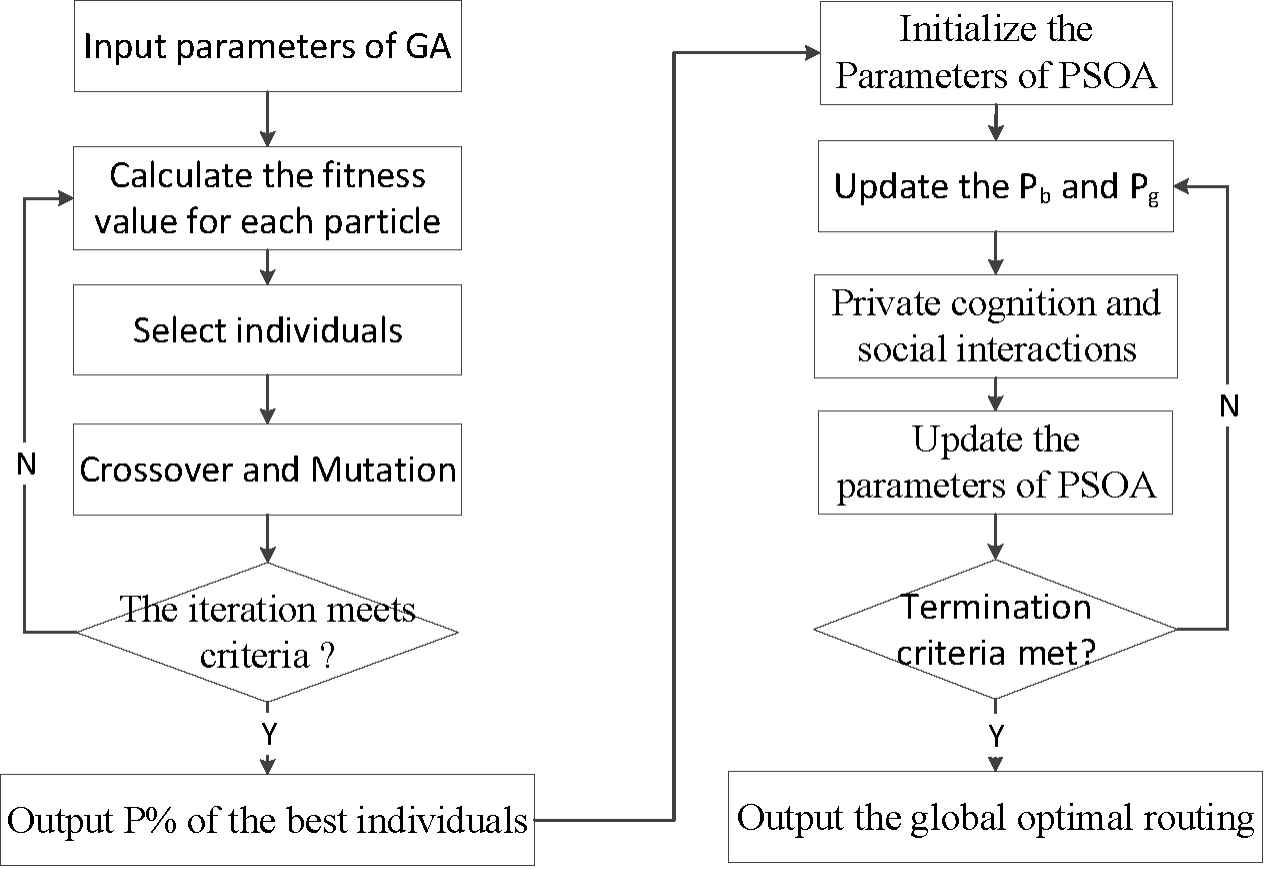
GA-PSOA computational flow chart.

The workflow of the GA-PSOA is shown in Fig 4.

In the process of GA-PSOA in the step 2, the fitness function *f*, the crossover probability *P*_*c*_ and the mutation probability *P*_*v*_ are shown as follows.

$$f=\frac{a}{T_{i}}+\frac{b}{E_{i}},$$ (1)

$$P_{c}=\left\{ \begin{array}{l} \frac{C_{1}(f_{\max}-f_{big})}{f_{\max}-f_{avg}}\;f_{big}\geq f_{\max}\\ C_{2}\;f_{big}<f_{avg} \end{array}\right.,$$ (2)

$$P_{v}=\left\{ \begin{array}{l} \frac{C_{3}(f_{\max}-f_{v})}{f_{\max}-f_{avg}}\;f_{big}\geq f_{\max}\\ C_{4}\;f_{big}<f_{avg} \end{array}\right..$$ (3)

In formula (1), *T*_*i*_, *E*_*i*_ denote time delay and the total energy consumption of all nodes in the *i* path respectively. *a* and *b* denote the adjustment proportion factors of fitness function which adapts the time delay and energy consumption. As the ZigBee nodes have low frequency of utilization and low power, most of the time it is in sleep mode, consuming very little energy. Hence, *a* is a little bit larger than *b*, which make contribute to a better performance of real-time. *f*_*max*_, *f*_*big*_, *f*_*agv*_, *f*_*v*_ denote the largest fitness value, the larger fitness value in crossover, the average fitness value, and the fitness value in mutations, respectively. *C*_*1*_, *C*_*2*_, *C*_*3*_, *C*_*4*_ are control factors, and the better individuals will be selected in the process of GA. Though the mutation may create the best individuals, the direction of the mutation cannot be controlled, and it tends to create a very large proportion of poor individuals. Taking the adaptation and diversity of the population into consideration, crossover operation hold a large probability, and mutation operation has a small probability.

Suppose the number of the excellent individuals selected by GA searching is M, they will be optimized by PSOA, and their original position is marked as the optimization solution. When they move in an *n*-dimensional space freely, they will search for the best position, and the other particles fly quickly in the space, and this information will be used to update the current optimization solution. The position and velocity of the *i-th* particle are denoted as *s*_*i*_ = (*s*_*i*1_, *s*_*i*2_,…, *s*_*in*_) and *v*_*i*_ = (*v*_*i*1_, *v*_*i*2_,…, *v*_*in*_), respectively.

During iteration, the optimization position of the *i-th* particle is *p*_*bi*_ = (*p*_*bi*1_, *p*_*bi*2_,…, *p*_*bin*_), and the optimization position of all particles in the particle swarm can be expressed as *P*_*g*_ = (*P*_*g*1_, *P*_*g*2_,…, *P*_*gn*_). All the particles will update their position and velocity according to Eq (4) and Eq (5).

$$v_{id}^{k+1}=\omega v_{id}^{k}+c_{1}\xi_{1}(p_{bid}-s_{id}^{k})+c_{2}\xi_{2}(p_{gd}-s_{gd}^{k})$$ (4)

$$s_{id}^{k+1}=s_{id}^{k}+v_{id}^{k+1},$$ (5)

where, *ω* denotes inertia weight, *ξ*_1_ and *ξ*_2_ are random numbers between 0 and 1, and *c*_*1*_, *c*_*2*_ denote the weights of the acceleration, which take the asymmetric linear forms:

$$c_{1}=c_{1s}+(c_{1e}-c_{1s})∙(i/i_{max}),$$ (6)

$$c_{2}=c_{2s}+(c_{2e}-c_{2s})∙(i/i_{max}).$$ (7)

In Eqs (6) and (7), *c*_*1s*_ and *c*_*2s*_ are the initial values of *c*_*1*_ and *c*_*2*_ respectively, and *c*_*1e*_ and *c*_*2e*_ are the final values of *c*_*1*_ and *c*_*2*_ in iterations.

In iterations, particles track the optimal solution and follow the optimal particle. These particles will update their position and velocity according to Eqs (4) and (5), and share the information about the optimal solution. In the preliminary, particles mainly find their own routes, then *c*_*1*_ will decrease and *c*_*2*_ will increase gradually, and begin to share information. Through multiple iterations, the globally optimal solution will be searched for and saved to the routing table. In iterations of PSOA, the optimal solution is transmitted in the one-way mode, which guarantees the particles rapidly move to the globally optimal direction. Further, it can solve the problem that the GA has slow convergence rate and may be trapped in local optimal solution.

### 3.3 The simulation of the routing algorithm in ZigBee network

**Fig 5:**
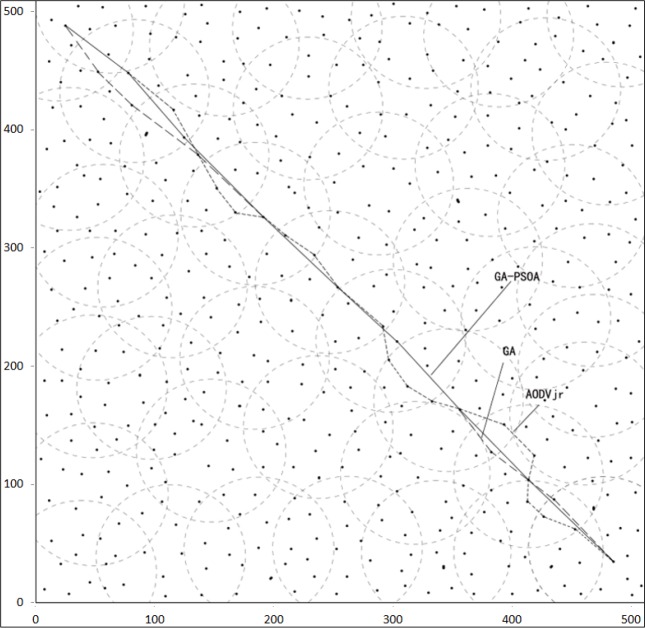
ZigBee network routing layout after simulation.

The simulative environment is as follows: Intel Core i5-2450M, 4G RAM; VMware Workstation 8.0 is installed in Windows7, and then Red Hat Enterprise Linux5. An extensive simulation in NS-2.34 and IEEE 802.15.4 PHY/MAC protocols is used to compare GA-PSOA with AODVjr and GA, where simulation parameters are as shown in Table 2. The source node will send one packet per second, and the length of each packet is 70 bytes. The coordinator node is powered by 3 Volt DC. The network routing layout of ZigBee nodes is as shown in Fig 5.

**Table 2:** Simulation parameters.

Simulation Parameters	Values
Deployment type	Random
Number of nodes	500
Number of coordinator	1
Number of router	99
Number of end devices	400
Voltage of nodes	3V
Initial energy of nodes	200J
communication distance	70m
Network dimension	500m×500m
PHY/MAC protocol	IEEE802.15.4
Routing algorithm	AODVjr/GA/GA-PSOA
Simulation time	180 minutes

**Fig 6:**
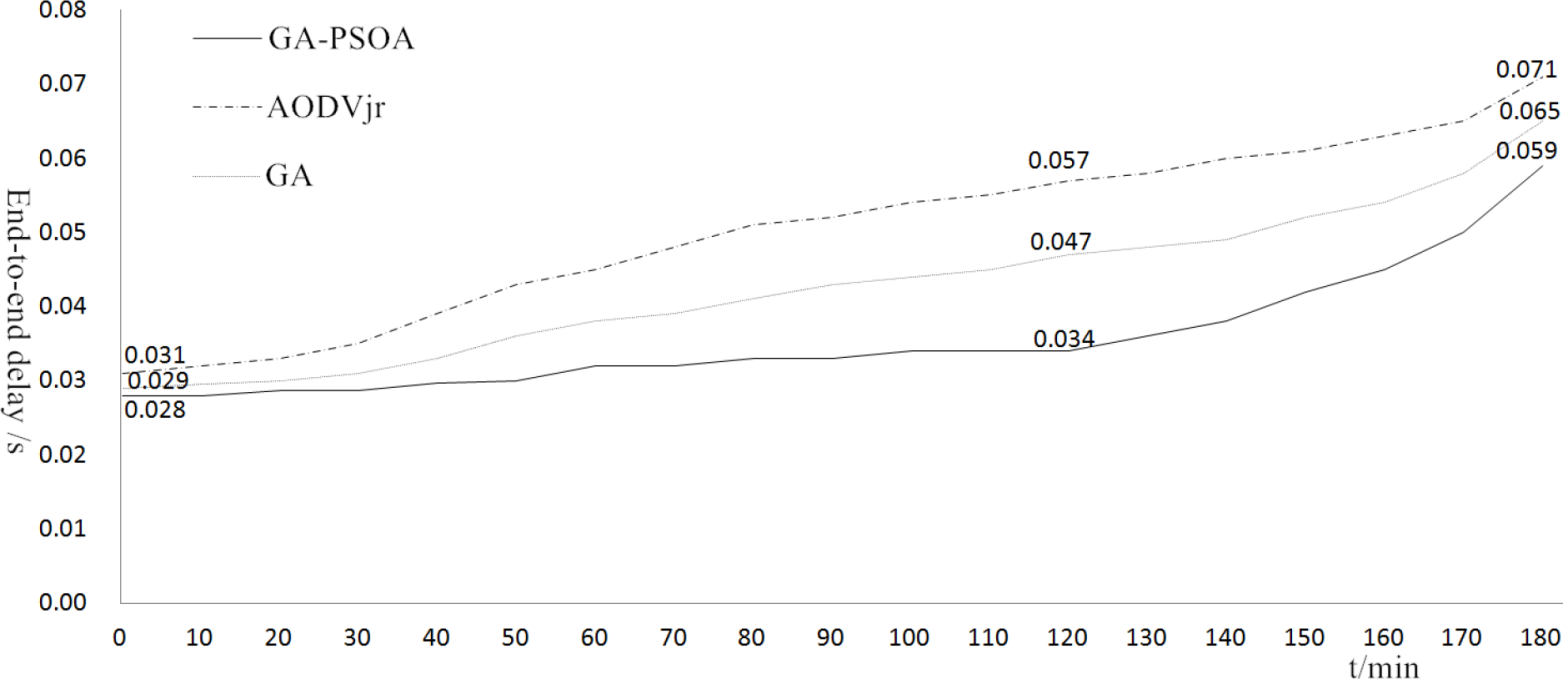
End-to-end delay.

The Fig 4 shows that the process of GA-PSOA contains two parts, GA and PSOA. Through an extensive simulation about the performance of the network topology in NS2, parameters are set up as follows. In the process of GA: *a* = *b* = 1, *c*_*1*_ = 0.95, *c*_*2*_ = 0.4, *c*_*3*_ = 0.02, *c*_*4*_ = 0.05, and the number of generations is 25. In the process of PSOA: *k* = 3.5, *ω* = 0.6, *c*_*1s*_ = 2.5, *c*_*2s*_ = 1.5. *c*_*1e*_ = 1.0, *c*_*2e*_ = 2.8, *i*_*max*_ = 15. The simulation of the end-to-end delay is recorded per minute. The result is as shown in Fig 6.

Fig 6 shows that the end-to-end delay of the three algorithms is similar during the first 30 minutes. However, GA-PSOA can keep a stable delay from 30 minutes to 120 minutes, and its value is smaller than the AODVjr by 0.023 at the 120th minute. After 180 minutes, the end-to-end delay with GA-PSOA is 0.059s, AODVjr and GA are 0.071s and 0.065s respectively. In this simulation, it is not obvious that the end-to-end delay of GA-PSOA is superior to GA, since GA can also search for the optimal route. However, if the route has one dead node, GA will need much more time to search for the next optimal route. Thus, GA-PSOA is superior to AODVjr and GA in the aspects of the end-to-end delay and the energy consumption.

## 4. The framework design of remote control

The smart home system provides users a convenient way to monitor and control smart home devices at any time, and the data collected by sensors or alarming information can also be sent to the remote control devices. In this part, we will design the remote system and discuss the experiment result.

### 4.1 Analysis of the communication in the smart home system

In the smart home system, users maybe monitor smart home devices at any time, and the data collected by sensors or alarming information needs to be send to the remote control devices. Thus, remote servers should be connected to the household router at any moment, providing a scalable and extensible framework, which is intuitive to new users, and allowing access from any internet connected device [20]. This system will be achieved by socket long connection mode.

**Fig 7:**
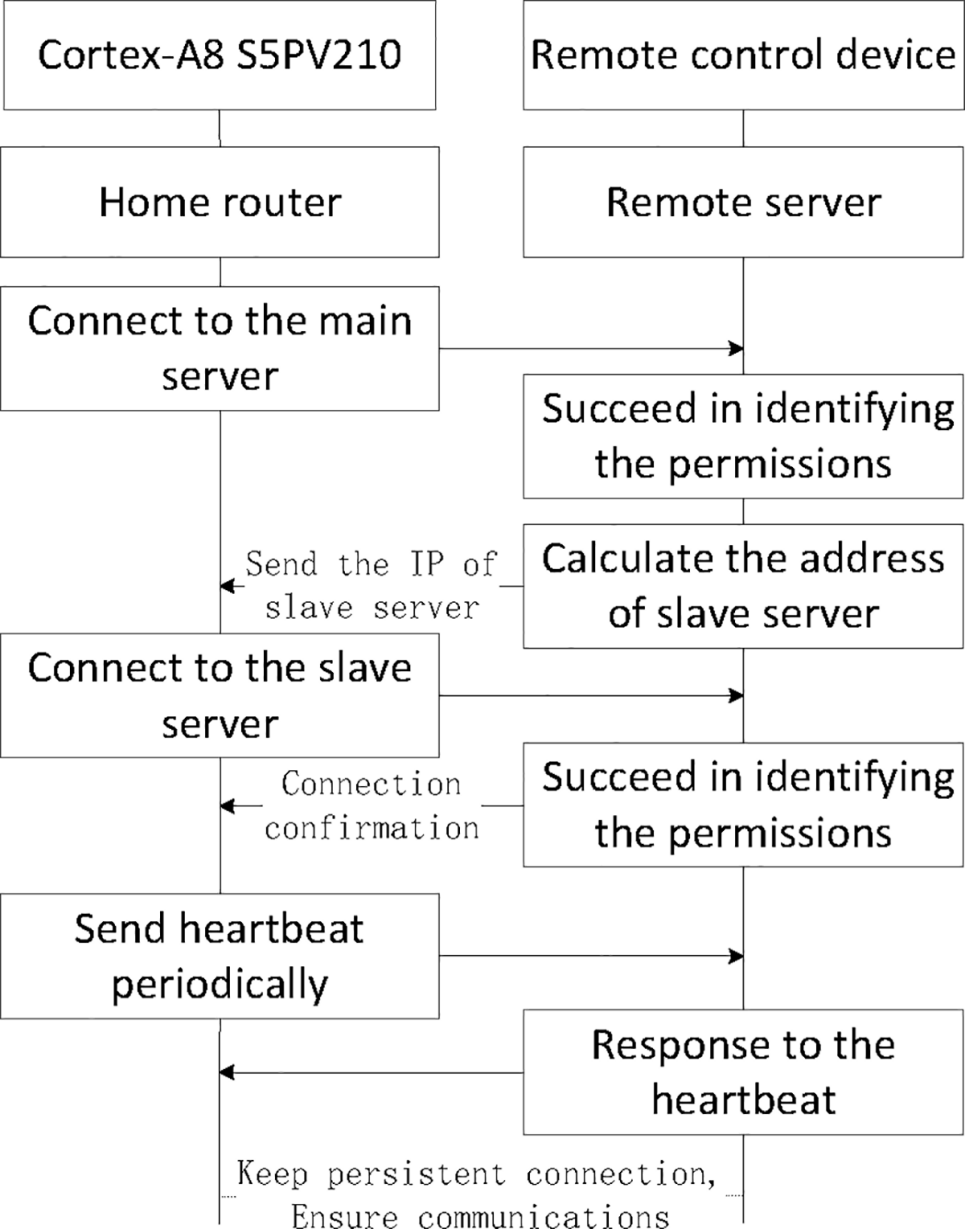
The server connection process.

When the connection is established, heartbeat package will keep it without interruption. The heartbeat interval is set to be thirty seconds, checked and the condition of the heartbeat connection. If the power is off or network is disconnected, the server will initiate connections until it returns to normal, and communicate with the household router by the socket connection. In order to improve the concurrency, throughput, and stability of the system, the remote servers is made of the master and slave servers in this system. On the one hand, the master server takes charge of setting up the first connection between the household router and the server, and at the same time, it can realize the dynamic load balance and avoid the cluster overload. One the other hand, the slave servers can keep the long connection with the household router by heartbeat package, which can ensure the real-time communications without interruption. The connection process of ZigBee wireless sensor network and the remote server is shown in Fig 7.

The master server acts as the balancer. Therefor it can deal with the cluster balancing and some existing load balance scheduling [21]. In this remote control system, the least connections algorithm works for the dynamic load balancing. The pseudo code of the algorithm is shown as *Algorithm* 1.

*Algorithm* 1:*LEASTCONNECTIONS*()

**Input**: A server set *S*.

**Output**: *AS*, The available and the least connections server.

**Function**: *Schedule*(*Si*), with input *Si* and output the server status.

         *Num(Si)*, with input *Si* and output the number of loading server.

**Begin**

  **For** each *i* in *S* **do**

    **IF** *Schedule*(*Si*) large than 0 **Then**

        **For** each *j* in (i+1,n) **do**

            **IF** *num(Si)>num(Si)* **And** *schedule(Si)> = 0*

              *i ← j*

            **End if**

      **Return** *Si*

  **End for**

  **Return** *Null*

**End**

The least connections algorithm can query the number of connections in slave servers. It will collect the load information every once in a while, and then the new connection request will be connected to the low load slave servers, resulting in the dynamic load balance instead of cluster overload.

### 4.2 The models’ design of the server

**Fig 8:**
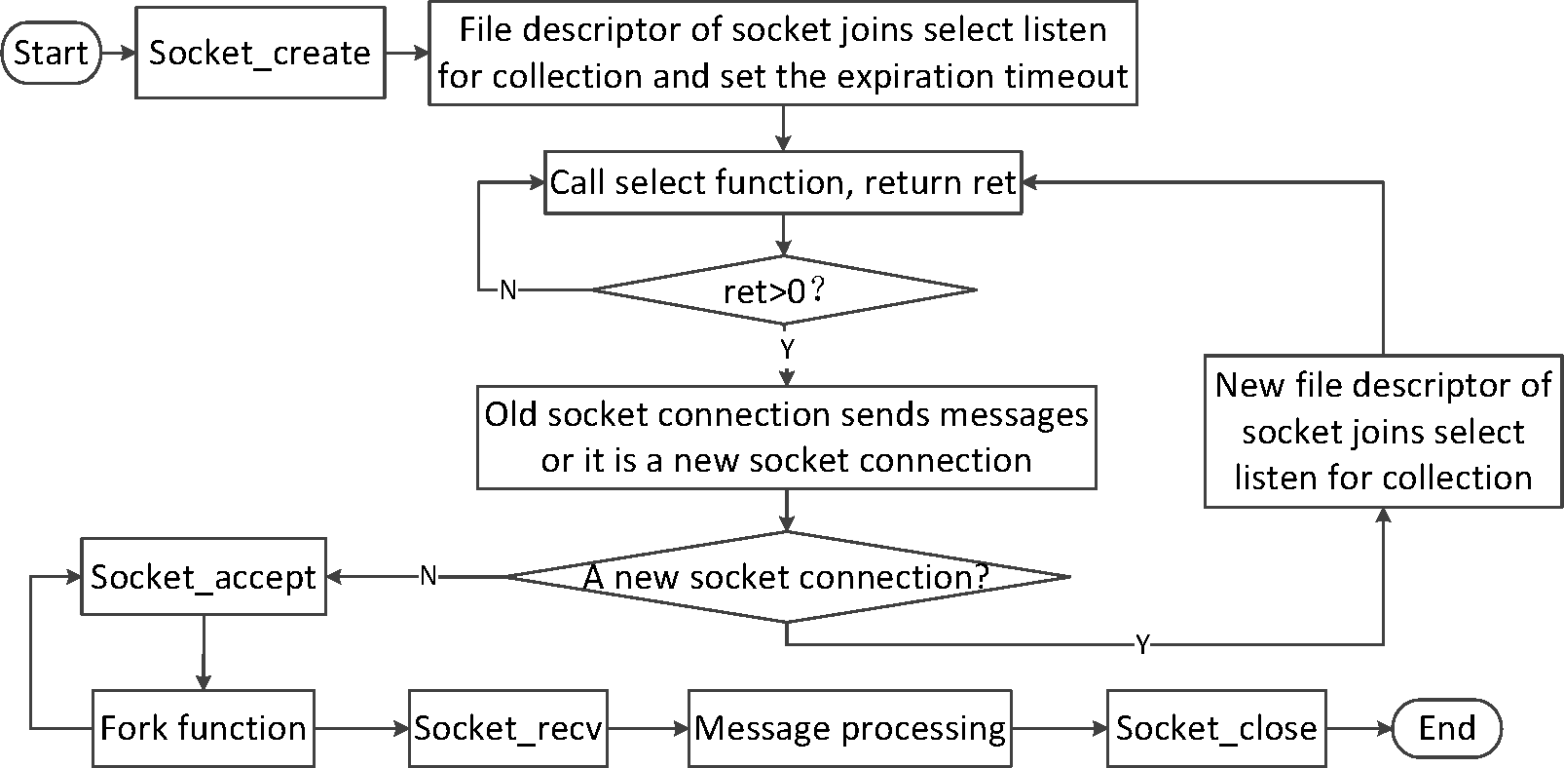
The server program workflow.

Through a survey of the state and the popularity of smart homes, right now the system of smart homes is not frequently used. However, with the development of society and the exaltation of our demand, there will be a great number of nodes joining the wireless sensor network. As a result, it will cause many security questions. For example, many nodes may visit the server at the same time, causing the data loss. The non-blocking multi-process servers are designed to overcome these problems. It applies the selecting technology to realize the I/O multiplexing, and builds a non-blocking multi-thread mode, which can handle multiple connection requests at the same time. Moreover, the fork function can be used to create sub-process, which can handle the multi-process [22]. The multi-thread and multi-process mode are shown in Fig 8.

When creating the socket server connection, the internet protocol address will bind the used port number, which is used to monitor the connection of remote control devices. To implement I/O multiplexing by the select technology, first, it will initialize the set of the file descriptor, which is monitored by the select. Second, the socket file descriptor is put into the select listening set, and the suitable maximum time of the connection request is set. Finally, the select mechanism is used to monitor multiple requests at the same time, which will build a non-blocking server. If the socket connections send multiple messages, the fork function can be used to create child processes. Furthermore, improving the performance, facility with multi-core CPUs will be the best choice to strengthen the processing capacity.

### 4.3 The program design of android application

**Fig 9:**
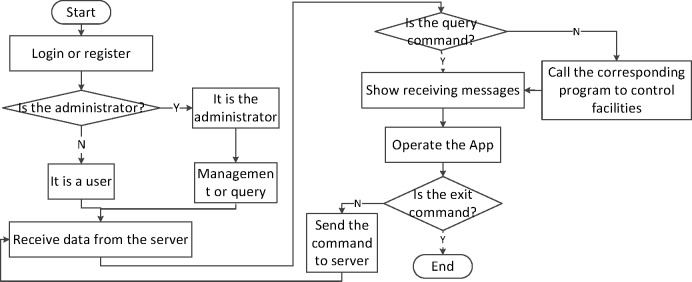
A flow chart from Android APP program.

An Android app is a good choice for the remote control device, by which the smart home system is monitored real-timely. Through this app, the Android mobile phones can keep communications with the remote servers to control the household devices and environment. The design of APP is shown in Fig 9.

If users log in to the app, they can manage the information, and real-timely monitor the smart home system. The system has set an access right, only the administrator can query and manage the information about users. Both administrators and users can receive the data from ZigBee network, and at the same time, they all can send control instructions on the terminal.

### 4.4 Results and discussion

In the smart home system, the basic function and performance are tested. The testing environment is as follows: it is Windows7 system in the testing computer, which is Intel Core i5-2450M, 4G RAM; the explorer is Mozilla Firefox; the system of Cortex-A8 S5PV210 is Linux; the ZigBee wireless mode uses Z-Stack protocol.

First, the basic functions such as monitoring the household environment, controlling the home devices, and the security system are tested. When the home router and all the nodes are turned on, the ability of building a ZigBee network will be tested, and then through the query instruction, what to test is whether the environment data can be sent to the app or not, such as the home temperature, and the state of lighting and doors. During the remote control, the status of the household devices can be seen in the app, and the control instruction will be sent to the ZigBee network, which tests whether the household device can make the corresponding operation or not, such as the control of the socket and magnetic door. In the security system, if the sensors are triggered, it will test whether the app can receive the alarm messages or not, such as someone burst into our house or production of smoke. The testing results of the basic functions are shown in Table 3.

**Table 3:** The basic functions test of smart home system.

Facilities	Test method	Test result
socket	Click the socket button	normal
lighting	Brightness Control	normal
temperature	Click the temperature button	normal
smoke	Release C_2_H_6_	alarm
infrared	through infrared sensors quickly	alarm
magnetic door	Click the magnetic door button	normal

**Fig 10:**
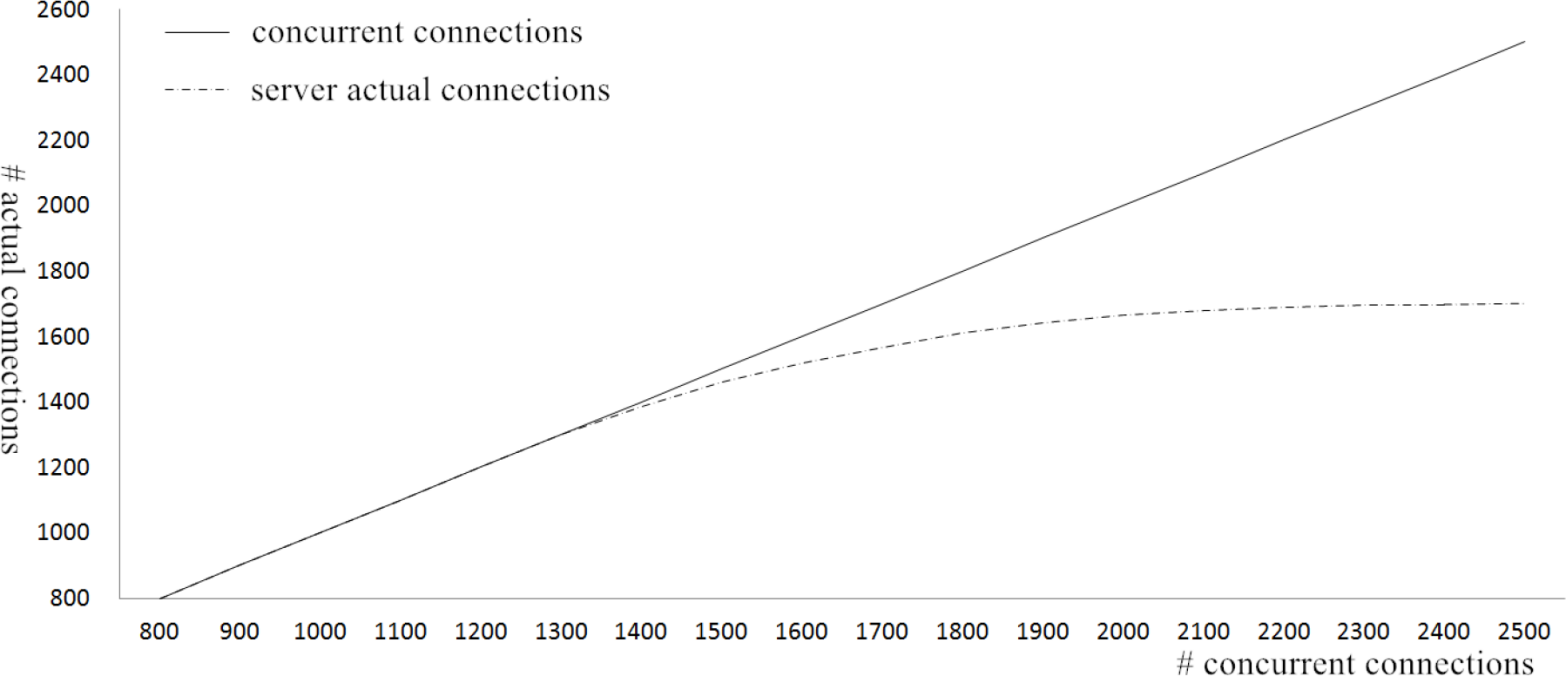
The performance testing of the server.

Experimental result shows that the system can remote control the facilities real-timely, and detect the home environment and state of the household electrics. If an exception occurs, it will automatically send an alarm to the user. To makes sure that the system can be used in a complex network and adapted to the rapid development of home environment, the practicality and performance needs to be tested. In this experiment, a large number of family gateways are simulated by the simulator, and they will request connections to the server, where the number of the initial connections is set as 400 and it will add the number of connections at the speed of 100 for every time. The performance testing result of the server is shown in Fig 10.

Through the extensive tests, results show that the server runs well if the number of request connections is less than 1300. Continue increasing the connections, messages would be lost, resulting in the situation where some connections cannot communicate with the server and the system will become unusual. In other words, the number of the connections with a server should be taken into consideration when a smart home system is to be designed. In this paper, the remote servers are composed of the master server and lots of slave servers, and the least connections algorithm is used on the master server, which will load much more connections through the remote server and realize the dynamic load balance in every slave server. In addition, high-performance servers can be used to design the remote smart home system, which will make the smart home system more perfect.

## 5. Conclusions

In the smart home system, first, household devices and sensor nodes successfully join the ZigBee network through the ZigBee technology, and GA-PSOA is used to search for the optimal route when the network is more complicated. Then the home wireless sensor network is connected to the remote server through the home router and the remote communication technology, which will create a platform for the remote control. Finally, the Android app has been designed, and the experimental test shows that the system can real-timely monitor the household environment and remote control the devices successfully.

In the future work, by memorizing the household environment and user’s habit, the big data mining technology, and personalized resources recommendation algorithm can be combined to realize the automatic and smart management of the smart home.
